# Donor Site Morbidity in Fibula Free Flaps: A Technique‐Dependent Comparative Analysis of Donor Site Wound Healing

**DOI:** 10.1002/micr.70139

**Published:** 2025-11-05

**Authors:** Jakob Fenske, Henri Kreiker, Philipp Lampert, Claudius Steffen, Steffen Koerdt, Susanne Nahles, Kilian Kreutzer, Max Heiland, Carsten Rendenbach, Norbert Neckel

**Affiliations:** ^1^ Department of Oral and Maxillofacial Surgery Charité—Universitätsmedizin Berlin, Corporate Member of Freie Universität Berlin and Humboldt‐Universität zu Berlin Berlin Germany

**Keywords:** donor site closure, fibula free flap, negative pressure wound therapy, primary closure, skin graft, two‐stage closure

## Abstract

**Background:**

The fibula free flap (FFF) is a mainstay in maxillofacial reconstruction, yet donor site morbidity remains a significant clinical concern. Closure technique is a key factor influencing complication rates, but comparative data remain heterogeneous. This study provides a technique‐dependent analysis of 60‐day donor site wound healing in FFF with skin paddles and reports a two‐stage closure approach.

**Methods:**

A retrospective review of 211 patients undergoing FFF between 2017 and 2024 was conducted. Donor site complications within the first 60 postoperative days were assessed and stratified by closure technique: one‐stage split‐thickness skin grafting (STSG) with or without negative pressure wound therapy (NPWT), and a two‐stage closure consisting of one‐week NPWT followed by STSG. Multivariate logistic regression was applied to identify independent predictors for complications.

**Results:**

Overall, 50.2% of patients experienced donor site complications, with wound healing disorders (31.8%) and (partial) skin necrosis (23.7%) most prevalent. Regarding wound closure, STSG coverage without NPWT was associated with the highest morbidity and a complication rate of 91.0%. Despite being limited in sample size, the two‐stage closure, despite larger skin defects, showed complication rates comparable to one‐stage closure with NPWT. Multivariate analysis identified STSG with NPWT (OR 0.1 [0.01; 0.4], *p* = 0.002) and two‐stage closure (OR 0.1 [0.01; 0.5], *p* = 0.01) as protective factors for wound healing complications.

**Conclusion:**

Donor site morbidity following FFF harvest with skin paddles is significantly impacted by closure technique. Wound management using one‐ or two‐stage STSG with NPWT is preferred. Initial results of the two‐stage closure indicate potentially beneficial outcomes for extensive defects and warrant further prospective validation.

## 1 Introduction

The fibula free flap (FFF) is a cornerstone in reconstructive surgery, particularly for extensive osseous defects of the maxillofacial region. Owing to its robust vascular pedicle, segmental bone availability, and potential for incorporating soft tissue components, the FFF offers versatility for composite reconstruction following trauma, oncologic resection, or osteonecrosis. However, while the success of the FFF at the recipient site has been well documented, morbidity at the donor site remains an important clinical consideration (Attia et al. 2020; Archibald et al. 2023; Momoh et al. 2011). Donor site complications, ranging from delayed wound healing and infection to tissue necrosis, can adversely impact recovery, prolong hospitalization, and affect long‐term functional outcomes such as gait and ambulation (Sieg et al. 2010; Kim et al. 2008; Denys et al. 2024). Consequently, closure techniques at the fibula donor site have become a focal point in efforts to mitigate postoperative morbidity, with identification of the most beneficial techniques still pending (Shimbo et al. 2022).

Several studies have examined factors influencing donor site morbidity, with particular attention to closure techniques including primary closure, skin grafting and application of negative pressure wound therapy (NPWT), as well as functional rehabilitation (Feng et al. 2020; Bach et al. 2016; Rendenbach et al. 2016, 2018). Moreover, different designs of free or pedicled local and regional flaps for wound closure have been proposed (Lee et al. 2025; Kaleem et al. 2021; Wang et al. 2016; Squadrelli‐Saraceno et al. 2010). Findings from existing literature suggest that donor site complications regularly occur and may be influenced by closure methods, skin paddle inclusion, and flap size, while patient‐specific risk factors such as comorbidities do not seem to play a pivotal role (Momoh et al. 2011; Shimbo et al. 2022; Feng et al. 2020). The benefit of external devices such as NPWT to improve wound healing, remains a subject of debate (Bach et al. 2016; Ho et al. 2013). Furthermore, our institution recently adopted a two‐stage closure for donor site defects with skin deficits based on the clinical idea that shear forces between scarcely free granulating muscle portions and STSGs may impair sufficient and timely healing. Therefore, donor sites initially receive NPWT for 1 week to promote granulation and are subsequently covered with a STSG and conventional wound dressing. Despite the variety of prior studies, comparative data on closure techniques remain heterogeneous, with wide variability in outcome definitions, follow‐up durations, and sample sizes, leaving clinicians with limited evidence to guide closure strategy selection. Additionally, the implementation and outcomes of two‐stage closure strategies have not yet been investigated.

To close this gap, this study aims to provide a comprehensive, technique‐dependent analysis of 60‐day donor site wound healing following FFF harvest with included skin paddles. By leveraging a large, single‐institution cohort, we seek to evaluate the impact of closure technique and timing on donor site complications, assess the role of skin paddle inclusion and size, and identify independent predictors of adverse outcomes. Additionally, we describe the two‐stage donor site closure. Thereby, this study aims to inform surgical decision‐making, optimize postoperative care, as well as derive translational insights from single‐center reflective surgical practices.

## 2 Materials and Methods

### 2.1 Study Cohort Selection

All patients undergoing FFF surgery for reconstruction of maxillofacial defects in the Department of Oral and Maxillofacial Surgery at Charité—Universitätsmedizin Berlin between April 2017 and December 2024 were filtered and identified. Exclusion criteria included (1) patients below the age of 18 (*n* = 0), (2) missing documentation data on donor site closure (*n* = 7), (3) FFF without additional skin paddles (*n* = 196), and (4) available postoperative follow‐up of below 60 days (*n* = 35). All remaining patients were included in the study cohort and deemed suitable for analysis. Institutional Review Board (IRB) approval was granted by the local ethics committee (EA2/138/18). Due to the retrospective and anonymized nature of this data analysis, individual patient consent was waived for this study. This study was performed according to the Declaration of Helsinki.

### 2.2 Study Variables

**FIGURE 1:**
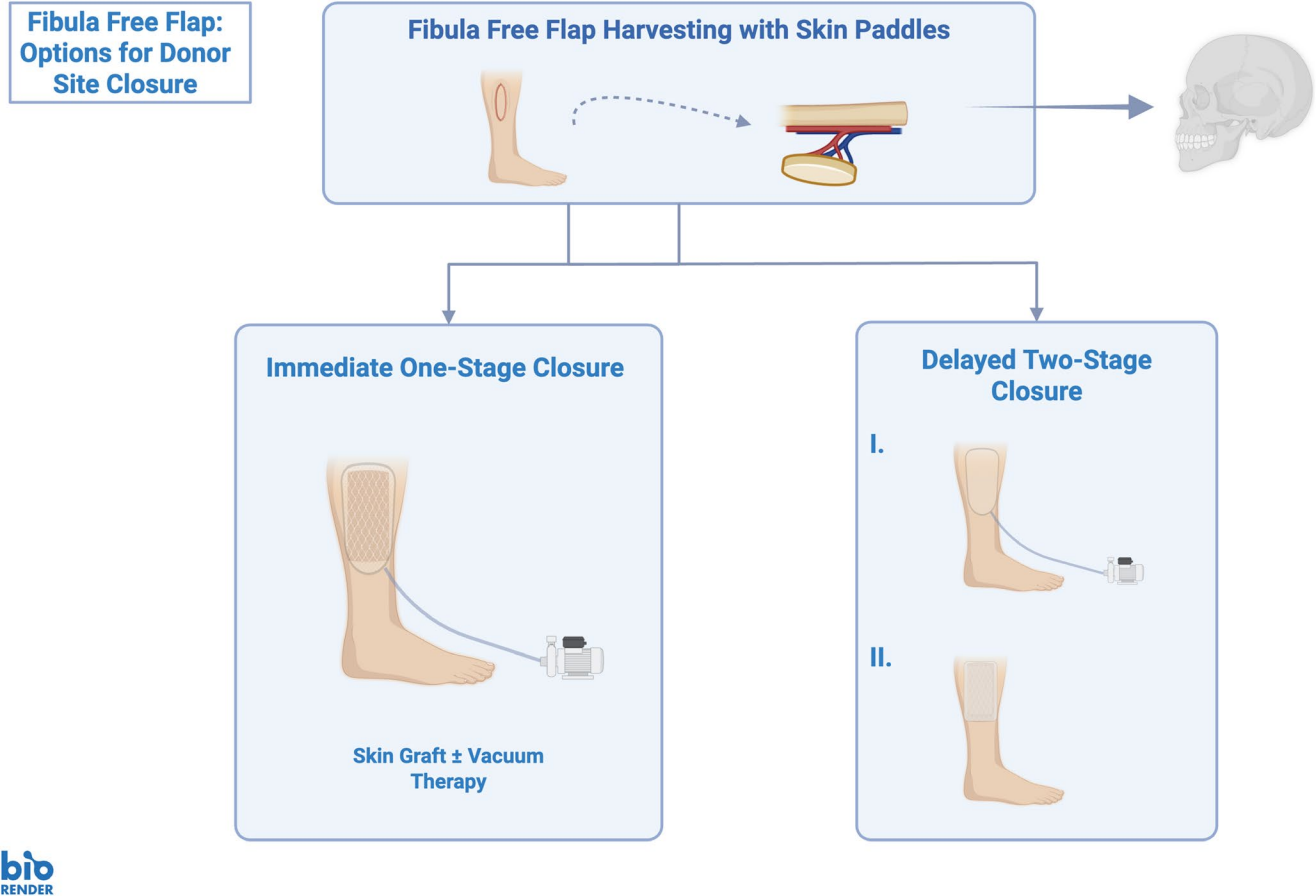
Overview of available closure options for fibula free flap donor sites including skin paddles.

Patient records were screened for baseline, predictor and outcome variables on donor site morbidity of FFFs. Evaluated variables included epidemiological data (age, sex, length of hospital stay, body mass index), comorbidities, donor site specifications (skin paddle size, donor site closure technique), follow‐up, and donor site wound complications (any complication, wound healing disorders [prolonged epithelialization or remaining wound dehiscence], wound infections [clinical infections requiring local antiseptics or antibiosis], [partial] skin necrosis [skin graft necrosis requiring debridement], and seroma [local donor site seroma formation]). Available closure techniques were one‐stage skin graft closure using split‐thickness skin grafts (STSGs) with or without NPWT and two‐stage closure using an initial NPWT dressing for 1 week followed by STSG with conventional wound dressing (Figure 1). The allocation to either closure technique was mainly due to intraoperative findings such as impossible primary closure due to tissue tension after skin paddle grafting. Closure using STSG without NPWT instead of STSG with NPWT was due to surgeon preference. The two‐stage closure was newly implemented for more recent cases and patients were allocated to this technique as a new standard in FFF harvest including skin paddles without further specification. Donor site complications occurring during the first 60 days following the initial free flap surgery were collected from follow‐up records. This cut‐off was determined, because 87.3% of complications occurred within the first 60 postoperative days (PODs) with a mean of 37.0 ± 26.2 days after surgery.

### 2.3 Data Management and Statistical Analysis

Data were collected and organized using spreadsheet software (Microsoft Excel 2024, Microsoft Corporation, Redmond, WA, USA). All statistical calculations were performed in RStudio for Mac (Version 2024.09.0 + 375). Descriptive statistics were reported using absolute and relative frequencies, as well as means and standard deviations. Exploratory subgroup analyses were performed for (1) one‐stage skin grafts with vs. without NPWT, and (2) one‐stage closure with NPWT vs. two‐stage closure procedures. Qualitative variables were compared using the *χ* ^2^ test, while metric variables were compared using the Wilcoxon rank sum test after assessing data distribution via the Shapiro–Wilk Test. Separate multivariate predictive models on significantly differing complications “any donor site complication” and “wound healing disease” were developed via binary logistic regressions using age, sex, two‐stage closure and one‐stage skin graft closure with NPWT as independent predictors. Inclusion of the null value in the 95% confidence interval (CI) of odds ratios (OR) was recorded as nonsignificant, while non‐inclusion was recorded as significant. A *p* value threshold of < 0.05 indicating statistical significance was adopted for the whole study. The level of evidence of the present study is classified as a Level IV Therapeutic Study (retrospective case series).

## 3 Results

### 3.1 Baseline Characteristics and Complication Rates of the Overall Cohort

A total of 211 patients (33.2% (*n* = 170) females) with a mean age of 62.1 ± 11.1 years were included in the study. All included patients had a full 60‐day follow‐up. The mean skin paddles size was 510 ± 37.4 cm^2^. Arterial hypertension (*n* = 82; 38.9%) and nicotine abuse (*n* = 84; 39.8%) were the most common comorbidities. Donor sites were mostly closed in a one‐stage approach (*n* = 194, 91.9%) with skin grafts with NPWT being the most common technique (*n* = 183; 94.3%), followed by skin grafts without NPWT (*n* = 11; 5.7%). If NPWT was used, a continuous pressure of −100 mmHg was applied for one week. No donor site was primarily closed. Patients were generally mobilized to the bedside starting on POD 1, allowed to stand on POD 2, to walk with a rollator on POD 3 and to climb stairs from POD 7 onwards under physiotherapeutic supervision. In total, 106 patients (50.2%) experienced any type of donor site wound complications. Specifically, wound healing disorders (*n* = 67; 31.8%), wound infections (*n* = 9; 4.3%), (partial) skin necrosis (*n* = 50; 23.7%), and seroma formation (*n* = 6; 2.8%) were observed. Table 1 details the baseline characteristics of the overall cohort.

**TABLE 1:** Baseline characteristics and complication rates of the total cohort. Results are presented as *n* (%) unless otherwise stated.

Variables	Total cohort (*N* = 211)
*Epidemiological data*	
Age (years ± SD)	62.1 ± 11.1
Female	70 (33.2%)
Length of hospital stay (days ± SD)	23.1 ± 14.6
Length of ICU/IMC stay (days ± SD)	2.9 ± 7.5
Body mass index (kg/m^2^ ± SD)	23.6 ± 4.3
*Comorbidities*	
Nicotine abuse	84 (39.8%)
Alcohol abuse	50 (23.7%)
Arterial hypertension	82 (38.9%)
Diabetes mellitus	18 (8.5%)
Arteriosclerosis	30 (14.2%)
Hyperlipidemia	20 (9.5%)
Hypothyroidism	29 (13.7%)
History of thrombosis	11 (5.2%)
Prior systemic chemotherapy	48 (22.7%)
*Donor site specifications*	
Skin paddle size (cm^2^ ± SD)	51.0 ± 37.4
*Donor site closure*	
One‐stage	194 (91.9%)
Two‐stage	17 (8.1%)
Time until second closure (days ± SD)	11.4 ± 4.9
Closure technique in one‐stage closure	
Skin graft with NPWT	183 (94.3%)
Skin graft without NPWT	11 (5.7%)
Maximum available follow‐up (months ± SD)	26.5 ± 21.9
*Donor site complications*	
Any complication	106 (50.2%)
Wound healing disorder	67 (31.8%)
Wound infection	9 (4.3%)
(Partial) Skin necrosis	50 (23.7%)
Seroma	6 (2.8%)

Abbreviations: ICU/IMC = intensive care unit/intermediate care unit, NPWT = negative pressure wound therapy, SD = standard deviation.

### 3.2 Subgroup Analyses on Complication Rates

Subgroup analysis of skin graft closure with and without NPWT showed elevated rates of any donor site complication (48.6% skin graft with NPWT vs. 91.0% skin graft without NPWT; *p* = 0.011) and wound healing disorders (29.0% vs. 81.8%; *p* = 0.0009) in skin grafts without NPWT, as detailed in Table 2.

**TABLE 2:** Subgroup analysis of donor site complications between skin grafts with and without NPWT in one‐stage donor site closures of fibula free flaps. Results are presented as *n* (%) unless otherwise stated. Statistically significant results are highlighted in bold.

Variables	One‐stage skin graft with NPWT (*n* = 183)	One‐stage skin graft without NPWT (*n* = 11)	*p*
*Epidemiological data*			
Age (years ± SD)	61.7 ± 11.2	64.5 ± 9.5	0.41
Female	62 (33.9%)	3 (27.3%)	0.90
Body mass index (kg/m^2^ ± SD)	23.6 ± 4.4	24.0 ± 3.5	0.51
*Comorbidities*			
Nicotine abuse	76 (41.5%)	3 (27.3%)	0.54
Alcohol abuse	46 (25.1%)	1 (9.1%)	0.40
Arterial hypertension	71 (38.8%)	5 (45.5%)	0.90
Diabetes mellitus	17 (9.3%)	1 (9.1%)	> 0.99
Arteriosclerosis	26 (14.2%)	2 (18.2%)	> 0.99
Hyperlipidemia	18 (9.8%)	1 (9.1%)	> 0.99
Hypothyroidism	28 (15.3%)	1 (9.1%)	0.90
History of thrombosis	11 (6.0%)	0 (0.0%)	0.87
Prior systemic chemotherapy	53 (29.0%)	1 (9.1%)	0.91
*Donor site specifications*			
Skin paddle size (cm^2^ ± SD)	46.4 ± 32.4	45.7 ± 36.6	0.99
*Donor site complications*			
Any complication	89 (48.6%)	10 (91.0%)	**0.02**
Wound healing disorder	53 (29.0%)	9 (81.8%)	**0.0009**
Wound infection	7 (3.8%)	2 (18.2%)	0.14
(Partial) Skin necrosis	44 (24.0%)	4 (36.4%)	0.58
Seroma	5 (2.7%)	0 (0.0%)	> 0.99

Abbreviations: NPWT = negative pressure wound therapy, SD = standard deviation.

One‐stage with NPWT and two‐stage donor site closure approaches did not exhibit statistically significant donor site complication rates. Skin paddles were significantly larger in two‐stage closures (101.8 ± 49.5 vs. 46.4 ± 32.4 cm^2^; *p* < 0.0001). In the two‐stage approach, donor sites initially received a −100 mmHg black‐sponge NPWT wound coverage after flap harvesting to promote granulation and subsequently received a STSG with conventional compressive wound dressing after 11.4 ± 4.9 days after the initial surgery. One patient was discharged before the second closure, which was performed in an outpatient setting. Table 3 details the results of the stage‐dependent analysis.

**TABLE 3:** Subgroup analysis of donor site complications between one‐stage closure with NPWT and two‐stage donor site closure of fibula free flaps. Results are presented as *n* (%) unless otherwise stated. Statistically significant results are highlighted in bold.

Variables	One‐stage closure with NPWT (*n* = 183)	Two‐stage closure (*n* = 17)	*p*
*Epidemiological data*			
Age (years ± SD)	61.9 ± 11.2	64.9 ± 11.3	0.35
Female	62 (33.9%)	5 (29.4%)	0.92
Body mass index (kg/m^2^ ± SD)	23.6 ± 4.4	22.8 ± 3.7	0.39
*Comorbidities*			
Nicotine abuse	76 (41.5%)	5 (29.4%)	0.47
Alcohol abuse	46 (25.1%)	3 (17.6%)	0.70
Arterial hypertension	71 (38.8%)	6 (35.3%)	0.98
Diabetes mellitus	17 (9.3%)	0 (0.0%)	0.39
Arteriosclerosis	26 (14.2%)	2 (11.8%)	> 0.99
Hyperlipidemia	18 (9.8%)	1 (5.9%)	0.92
Hypothyroidism	28 (15.3%)	0 (0.0%)	0.17
History of thrombosis	11 (6.0%)	0 (0.0%)	0.63
Prior systemic chemotherapy	44 (24.0%)	2 (11.8%)	0.40
*Donor site specifications*			
Skin paddle size (cm^2^ ± SD)	46.4 ± 32.4	101.8 ± 49.5	**< 0.0001**
*Donor site complications*			
Any complication	89 (48.6%)	7 (41.2%)	0.74
Wound healing disorder	53 (29.0%)	5 (29.4%)	> 0.99
Wound infection	7 (3.8%)	0 (0.0%)	0.90
(Partial) Skin necrosis	44 (24.0%)	2 (11.8%)	0.40
Seroma	5 (2.7%)	1 (5.9%)	> 0.99

Abbreviations: NPWT = negative pressure wound therapy, SD = standard deviation.

### 3.3 Multivariate Models

In multivariate analysis, two‐stage closure (OR 0.1 [0.01; 0.5]; *p* = 0.02), and one‐stage closure with NPWT (OR 0.01 [0.01; 0.5]; *p* = 0.03) were associated with lower rates of any type of donor site complication, while age (OR 1.0 [0.9; 1.0]; *p* = 0.45) and sex (OR 0.6 [0.3; 1.0]; *p* = 0.07) did not show significant influences. Two‐stage closure (OR 0.1 [0.01; 0.5]; *p* = 0.01) and one‐stage closure with NPWT (OR 0.1 [0.01; 0.4]; *p* = 0.002) were independent predictors for lower wound healing disorders, with age (OR 1.0 [0.9; 1.0]; *p* = 0.35) and sex (OR 0.6 [0.3; 1.2]; *p* = 0.14) lacking significant influences.

## 4 Discussion

Reaching a balance between reconstructive success and minimal donor site morbidity is a defining challenge in free flap surgery. This study presents a comprehensive, technique‐dependent analysis of 60‐day donor site morbidity following FFF harvest, focusing on closure strategies and their outcomes in FFF with skin paddles. In a cohort of over 200 patients with complete follow‐up, critical associations between closure technique and postoperative complications were identified. Notably, our findings confirm that one‐stage closure without NPWT is associated with significantly more complications, while adjunctive strategies involving NPWT or two‐stage approaches offer measurable benefit. Additionally, the two‐stage donor site closure approach was introduced, showing preliminary promising results to potentially serve as a future avenue for managing complex donor sites.

The overall complication rate of 50.2% in this study slightly exceeds the spectrum of previously reported donor site morbidity following FFF harvest, where rates range between 20% and 35% depending on definitions, follow‐up duration and evaluated outcomes, such as nerve injuries or severe functional restrictions. Wound healing disorders and (partial) skin necrosis presented as the most common adverse outcomes. However, most of these studies encompassed substantially smaller cohorts and included FFF cases without additional skin paddles (Xiang et al. 2024). In detail, Momoh et al. (2011) reported complication rates of 31.2% in a cohort of 157 patients, closely approximating the results of this study, while Zimmermann et al. (2001) reported slightly higher rates with 38.1%. A systematic review by Ling and Peng (2012) found donor site complication rates of up to 19%, although complication types were stratified in many different categories. Interestingly, while infectious complications are generally rare, the wound infection rate of this study (4.3%) aligns with the lower spectrum of previous studies, where rates of 1%–10% were reported (Archibald et al. 2023). Furthermore, it must be noted that all reported complications occurred during the first 60 postoperative days. While this may lead to underreporting of a few complications, the adopted time frame seems justified, as the vast majority of complications occurred within this period. Thereby, statistical homogenization and full follow‐up for all included patients was ensured, increasing the study's rigor and providing a snapshot of clinically relevant morbidity. Importantly, all included cases involved skin paddles, that prevented primary closure and likely led to higher complication rates than primarily closed wounds, reinforcing the understanding that larger soft tissue harvests entail greater wound management challenges. Future efforts are warranted to further decrease complication rates by adhering to proven wound closure techniques and further validating them in prospective trials.

In this study, the analysis focused exclusively on donor site wound management in FFF harvested with skin paddles, preventing primary closure. The primary closure technique is generally known to be associated with low complication rates and has been extensively studied: Feng et al. identified primary closure as beneficial compared to skin graft coverage (Feng et al. 2020). Of note, Akashi et al. (2016) found no difference between primary closure and skin graft coverage in a comparative study of 35 patients. However, primary closure is not always feasible, particularly in cases where large skin paddles are harvested, such as extensive intermaxillary defects or additional large mucosal deficits in the floor of the mouth, the tongue or the buccal plane. In such scenarios, STSGs become necessary for donor site closure. Among skin‐grafted wounds, those treated without NPWT, although limited in size, showed significantly worse outcomes than those managed with it in an exploratory approach. While some authors did not recommend the routine use of NPWT devices in FFF donor sites (Ho et al. 2013), our results are consistent with previous literature suggesting the adjunctive benefits of NPWT in improving graft take, stabilizing the wound environment, promoting wound healing pathways and managing exudate (Bach et al. 2016; Normandin et al. 2021; Gefen et al. 2024). In contrast, wounds closed with STSGs alone without NPWT experienced a substantially higher complication rate, indicating that this approach may be inadequate for optimal donor site healing.

Moreover, this study reports outcomes of a two‐stage donor site closure approach, introduced as a technique for managing extensive donor site defects, especially in cases involving large skin paddles. While the cohort undergoing this protocol is limited yet, they demonstrated significantly larger skin defect sizes but comparable or lower complication rates than those undergoing a one‐stage closure, despite using a conventional compressive wound dressing in the second stage. In multivariate analysis, two‐stage closure was an independent predictor for fewer wound healing disorders and overall complications. However, these findings must be interpreted under the limitation of the small sample size and wide CIs. The rationale for the two‐stage technique is based on deferred closure in cases of high tension or suboptimal tissue condition, allowing time for edema resolution, tissue conditioning, or delayed reconstruction with improved vascularity, as known from other specialties (Ceilley et al. 1983; Lewis et al. 2020; ElHawary et al. 2023). Importantly, the delayed closure also allows for granulation of the initially regularly exposed tendon of the fibularis longus muscle. Thereby, tissues receive time for sufficient granulation and wound ground preparation to facilitate healing and adaptation of the STSG in a second stage. A disadvantage of this technique is that patients are sometimes discharged between the two surgeries, leading to this approach being more sensitive to patient adherence. However, this circumstance did not translate into elevated complication rates in the presented results and was only relevant for 1 of 17 patients. Furthermore, another advantage of this technique is that the second stage closure is principally feasible under local anesthesia, thereby preventing risks of another general anesthesia and increasing patient acceptance. Thereby, the surgery can be performed outside of normal operation room schedules because no additional personnel from the anesthesia department is needed. Additionally, no prolonged hospital stay is provoked by the two‐stage closure, as it is typically performed within the normal hospitalization period, thereby staying in the common time frame indicated by public health gratification systems such as the diagnosis‐related groups (DRG). Consequently, while specific cost analyses are pending, our current experience indicates no increased financial burden. Our preliminary findings suggest that this approach seems to be comparably safe, effective, and may be beneficial for larger defects. Though limited by sample size, the two‐stage method may offer a flexible and promising solution for challenging donor sites that cannot be closed primarily or where primary grafting carries elevated risk. Further exploration in larger, multicenter cohorts is warranted to validate its efficacy and establish standardized protocols.

Based on these findings, the following options for a stratified wound closure approach seem to be clinically feasible: if primary closure is not possible due to skin paddle inclusion, STSGs with NPWT as a one‐or two‐stage procedure are recommended to improve outcomes and reduce healing complications. The current evidence also suggests, that especially larger skin defects benefit from a two‐stage closure approach. Future research should incorporate prospective, randomized studies directly comparing closure techniques to validate these retrospective findings, as well as the possibility to also use NPWT in the second stage in two‐stage to further improve healing. Moreover, the role of patient‐reported outcomes, functional and aesthetic outcomes should be integrated into future morbidity assessments.

## 5 Limitations

This study is limited primarily by its retrospective design, which introduces inherent selection biases and limits control over confounding variables. Although multivariate analyses were used to adjust for several key factors, residual confounding cannot be excluded. Second, the sample sizes for certain subgroups, especially the two‐stage closure and STSG without NPWT groups, were relatively small, limiting the generalizability of those specific findings and leading to a rather exploratory data interpretation. Third, while the 60‐day follow‐up period was appropriate for capturing early morbidity, it does not account for other complications such as chronic pain, scarring, or gait abnormalities. Fourth, this analysis does not account for primary closed FFF without skin paddles and, although rarely performed, primary closed FFF with included small skin paddles. Fifth, no functional orthopedic analysis, such as impaired gait or joint mobility, of the lower limb and ankles was performed, limiting conclusions on functional rehabilitation. Finally, all data were derived from a single high‐volume institution, and practices may vary across different centers, potentially affecting external validity.

## 6 Conclusion

This single‐institutional study provides evidence that donor site morbidity following FFF harvest with inclusion of skin paddles is significantly influenced by closure technique. STSG closure in combination with NPWT in a one‐stage procedure emerged as the preferred method, offering the comparably lowest complication rates. One‐stage skin grafting without NPWT should be avoided due to unacceptable complication rates. The initial results of the newly introduced two‐stage closure approach, although limited by sample size, indicate potentially beneficial outcomes in cases with large skin paddle harvests, that warrant further prospective investigation. These findings contribute to optimizing donor site management in FFF surgery and lay the groundwork for future prospective studies aimed at standardizing closure protocols to improve patient outcomes.

## Author Contributions

Conceptualization: J.F., N.N., C.R. Methodology: J.F., N.N. Formal analysis: J.F. Investigation: J.F., H.K., P.L. Resources: M.H., C.R. Data curation: J.F., H.K., P.L. Visualization: J.F. Supervision: C.R., N.N. Project administration: N.N. Writing – original draft: J.F. Writing – review and editing: J.F., H.K., P.L., C.S., S.K., S.N., K.K., M.H., C.R., N.N.

## Ethics Statement

IRB approval was granted by the local ethics committee at Charité—Universitätsmedizin Berlin (EA2/138/18).

## Conflicts of Interest

The authors declare no conflicts of interest.

## Data Availability

The data that support the findings of this study are available on request from the corresponding author. The data are not publicly available due to privacy or ethical restrictions.
